# The Association and Mediating Role of Choroid Plexus Enlargement Between Cerebrospinal Fluid Inflammatory Markers, Amyloid Burden, and Cognitive Decline Across the Clinical Spectrum of Alzheimer's Disease

**DOI:** 10.1002/alz.091949

**Published:** 2025-01-09

**Authors:** Yoon Seong Choi, Pek‐Lan Khong, Calvin Tjio Kai En

**Affiliations:** ^1^ Clinical Imaging Research Center, National University of Singapore, Singapore Singapore; ^2^ National University Hospital, Singapore Singapore

## Abstract

**Background:**

The choroid plexus plays an important role in the pathophysiology of Alzheimer's disease (AD) and in immune surveillance. Neuroinflammation is known to occur in the early stages of AD, where amyloid accumulation leads to cognitive decline in later stages. However, the relationship between choroid plexus volume (ChPV) and cerebrospinal fluid (CSF) inflammatory markers, amyloid burden and cognitive decline in vivo remains underexplored. This study aims to investigate the association and mediating role of ChPV with 1) CSF inflammatory markers and amyloid beta 42 (Ab42); 2) Centiloids from amyloid PET and cognitive decline across the AD clinical spectrum.

**Method:**

For Aim 1), 345 participants from ADNI 1 with baseline MRI and CSF inflammatory markers (n=345 with complement 3 [C3] and factor H [FH] and n=295 with tumour necrosis factor receptor 1 [TNFR1]) were enrolled. For Aim 2), 1,202 participants from ADNI GO/2/3 with baseline MRI and amyloid PET were enrolled. The association and mediating role of ChPV with CSF inflammatory markers and CSF Ab42, and with centiloids and the Preclinical Alzheimer Cognitive Composite (PACC) were assessed using linear regression and causal mediation analysis. The association between ChPV and centiloids was also evaluated in an independent cohort (n=1,772).

**Result:**

ChPV showed a significant association with CSF inflammatory markers (P<0.001 for C3, P=0.001 for FH and TNFR1). ChPV mediated the relationship from CSF inflammatory markers to CSF Ab42 with significance or borderline significance (proportion mediated, 28.1%, 19.3% and 11.3%; P=0.014, 0.009 and 0.077 for C3, FH and TNFR1, respectively). ChPV showed a significant association with centiloids in the amyloid‐positive subgroup (P=0.008), with an interaction with amyloid‐positive status (P=0.009) in the overall study population. ChPV significantly mediated the association between centioids and PACC (proportion mediated, 3.2%, P=0.018). The association between ChPV and centiloids was replicated in an independent dataset (P=0.014).

**Conclusion:**

ChPV was associated with CSF inflammatory markers, and amyloid burden from CSF and PET. ChPV also mediated the effect from CSF inflammatory markers to amyloid burden and from amyloid burden to cognition.

